# Water-Soluble *N*-Acetyl-L-cysteine-Capped CdTe Quantum Dots Application for Hg(II) Detection

**DOI:** 10.1155/2013/902951

**Published:** 2013-07-09

**Authors:** Tianming Yang, Qingyi He, Yuying Liu, Chaozhen Zhu, Dan Zhao

**Affiliations:** College of Pharmacy, South-Central University for Nationalities, Wuhan 430074, China

## Abstract

A simple, rapid, and specific method for Hg(II) detection has been proposed based on the fluorescence change of *N*-acetyl-L-cysteine-capped CdTe quantum dots (QDs). The presence of Hg(II) ions could quench the fluorescence of QDs at 565 nm and meanwhile produce new peak in 700–860 nm wavelength range. The linear response range is 20–430 nM with the detection limit at 8.0 nM Hg(II). It was found that the position of the new peak was irrelevant to the size of QDs. Furthermore, the mechanism of the quenching of QDs fluorescence by Hg(II) and the appearance of new peak in near-infrared area were also discussed and deduced through ultraviolet absorption spectrum, fluorescence spectrum, and X-ray photoelectron spectrum.

## 1. Introduction

As a new class of potential fluorescence probes, quantum dots (QDs) have attracted great interests of the researchers because of their unique and excellent properties over traditional fluorescent dyes and fluorescent proteins [1–3]. Compared to conventional organic fluorescent dyes, QDs possess higher photoluminescence (PL), excellent quantum yield (QY), size-dependent tunable luminescence wavelength, wide continuous absorption, narrow fluorescence band, and better photostability. Over the past two decades, great efforts have been focused on the development of sensors [4–8] based on QDs, and the detection of metal ions is the active field. Some researchers have realized the specific detection of metal ions through modification of QDs with different surface-attached ligands [9–13], such as the detection of Cu^2+^ ions through thioglycerol-capped CdS QDs [9] and mercaptopropionic acid-coated core/shell CdTe/CdSe QDs [10], the detection of Zn^2+^ ions through L-cysteine-capped CdS QDs [9], the detection of Ag^+^ ions through thioglycolic acid-coated CdSe QDs [11], the detection of Cu^2+^ and Ag^+^ ions through peptide-coated CdS QDs [12], and the detection of Pb^2+^ ions through glutathione-capped ZnCdSe and CdTe QDs [13].

As one of the most toxic heavy metals and persistent contaminants which cannot be biodegraded in ecosystem [14, 15], mercuric ion (Hg^2+^) requires new and efficient detection methods. The major challenges in developing QDs-based Hg probe are the preparation of water-soluble QDs with high luminescence quantum yield and the selectivity of the system [16–19]. Herein, through hydrothermal route, a series of high-quality *N*-acetyl-L-cysteine- (NAC-) capped QDs with excellent water solubility, stability, and high QY (the average QY is 50%) have been synthesized [20–22]. Based on the prepared NAC-capped CdTe QDs as the fluorescence probe, a rapid, cost-efficient, sensitive, and selective detection method for Hg(II) ions has been developed in which Hg(II) efficiently quenches the fluorescence of QDs and produces a new peak in near-infrared area. Since size effect is a basic characteristic of semiconductor nanocrystals, the impact of particle diameter of QDs upon the system was also studied. The proposed Hg(II) detection mechanism was also deduced through fluorescence spectrum, ultraviolet absorption spectrum, and X-ray photoelectron spectrum (XPS). 

## 2. Experimental

### 2.1. Chemicals

Tellurium (reagent powder, 99.8%) and NAC were purchased from Sigma. CdCl_2_ *·*H_2_O, Hg (ClO_4_)_2_, Rhodamine 6G, and sodium borohydride (NaBH_4_) were obtained from Aldrich. Deionised distilled (DI) water prepared from a Milli-Q-RO4 water purification system (Millipore) was used and purged with nitrogen (N_2_) for 30 min before use. All reagents were of analytical grade or above unless otherwise stated.

### 2.2. Synthesis of NAC-Capped CdTe QDs 

NaBH_4_ was used to react with tellurium with a molar ratio of 2 : 1 in DI water to prepare sodium hydrogen telluride (NaHTe). Fresh NaHTe solutions were then diluted by N_2_-saturated water for further use. CdCl_2_ (1.25 mM) and NAC (1.56 mM) were dissolved in 40 cm^3^ of DI water in an ice-water bath. The precursor solution was adjusted to pH 9 by stepwise addition of 1.0 M NaOH at 4°C. Subsequently, a fresh NaHTe solution at 0°C was added to the above prepared precursor solution and stirred vigorously. The molar ratio of Cd : Te : NAC was fixed at 1.0 : 0.2 : 1.2. Finally, the solution was put into a 40-cm^3^ Teflon-lined stainless steel autoclave. It was loaded in an oven at 200°C for a specified time (30–50 min) and then cooled to the room temperature by a hydrocooling process.

To remove NAC-Cd complexes at the end of the synthesis, cold 2-propanol was added to the reaction mixture to precipitate NAC-capped CdTe QDs. The QDs were dissolved in water and precipitated again with cold 2-propanol. The as-prepared products were dried overnight under vacuum at 40°C for further experiments. 5.0 *μ*M of CdTe QDs in 50 mM Tris-HCl buffer at pH 7.8 was used, and the excitation wavelength was 450 nm. The QYs of CdTe QDs were measured according to the literatures [23]. Rhodamine 6G in ethanol was chosen as the reference standard (QY = 95%).

### 2.3. Characterisation

UV-visible absorption spectra were acquired with a Varian Cary 100 Scan UV/visible spectrophotometer. Photoluminescence spectra were recorded on a Photon Technology International QM4 spectrofluorometer equipped with a thermoelectrically cooled InGaAs photodiode for near-infrared (NIR) region measurement. All PL spectra were corrected for spectral response of the detection optics. XPS measurements were acquired with a Leybold Heraeus SKL 12 X-ray photoelectron spectrometer.

## 3. Results and Discussion

**Figure 1:**
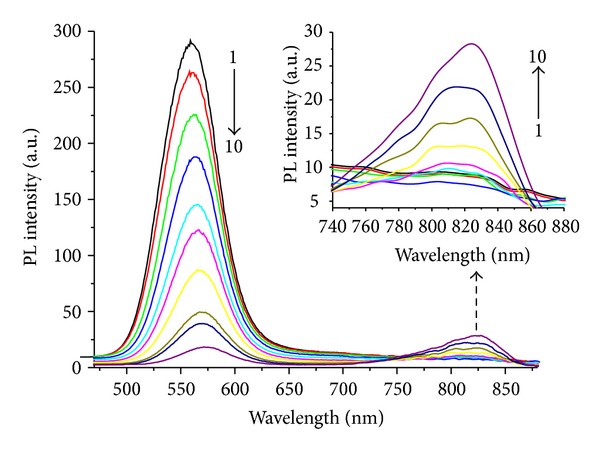
Effect of Hg(II) ions on the PL spectra of CdTe QDs (*λ*_em_ = 565 nm) with the concentration of Hg^2+^ at 0.0, 0.2, 0.5, 1.0, 2.0, 3.0, 4.3, 6.0, 8.0, and 10.0 × 10^−7^ M (from 1 to 10). The inset depicts the emergence of an NIR emission band on the addition of Hg(II) ions.

Figure 1 shows the fluorescence spectra of NAC- capped CdTe QDs (*λ*_em_ = 565 nm) in Hg(II) ions titration experiments (in 50 mM Tris-HCl buffer at pH 7.8). As shown in Figure 1, Hg(II) ions can efficiently quench the PL intensity of CdTe QDs. With the addition of Hg(II) ions, the PL peak intensity at 565 nm decreases with a slight bathochromic shift of PL spectrum. When Hg(II) ions concentration is increased to 1.0 *μ*M, the PL peak intensity reduces to only a few present of its original value. Meanwhile, new emission peak appears at 700–900 nm, and its fluorescent intensity increases with the addition of Hg(II) ions (inset of Figure 1). 

**Figure 2:**
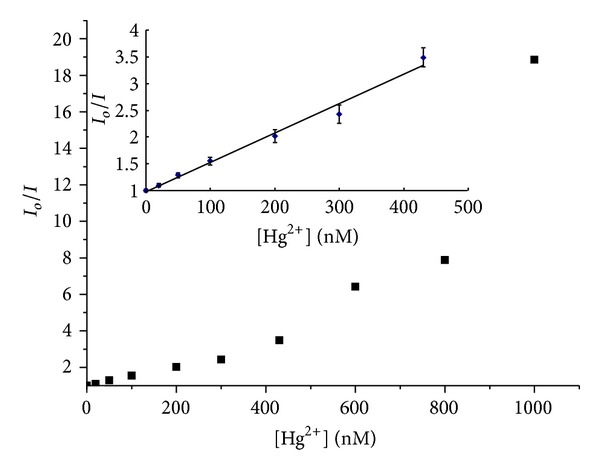
Stern-Volmer relationship between PL intensity of CdTe QDs and Hg(II) ions. The inset displays a linear Stern-Volmer plot at the low concentration range of Hg(II) ions with their error bars.

The relationship between fluorescence intensity of CdTe QDs and concentration of Hg(II) ions can be described by Stern-Volmer plot as follows:

(1) $$\begin{array}{c} \frac{I_{o}}{I}=1+K_{\text{sv}}\left[Q\right], \end{array}$$

where *I*_*o*_ and *I* are the PL intensities of CdTe QDs in the absence and presence of quencher *Q*, [*Q*] is the Hg(II) ion concentration, and *K*_sv_ is the Stern-Volmer constant.  Figure 2 describes a Stern-Volmer quenching curve with *I*_*o*_/*I* as a function of Hg(II) ion concentration, and a very good linearity is observed in the lower concentration range. The linear rang is from 20 to 430 nM with *K*_sv_ at 5.49 × 10^6^ M^−1^. The limit of detection is 8.0 nM which is determined on the basis of three times the standard deviation of six replicate measurements of the quenched PL intensity by the addition of 30 nM Hg(II). However, when the concentration of Hg(II) ions is higher than 430 nM, the line is curved upward because of superquenching effect (*vide infra*).

**Figure 3:**
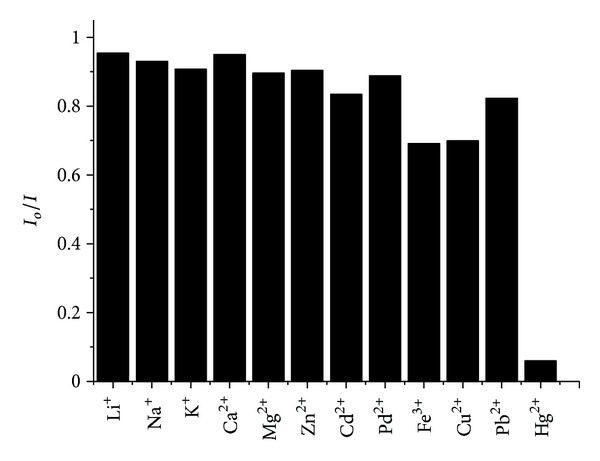
Effect of other metal ions on the PL of NAC-capped CdTe QDs (*λ*_em_ = 565 nm). The concentrations of Li^+^, Na^+^, K^+^, Ca^2+^, Mg^2+^, and Zn^2+^ were 1 × 10^−3 ^M. The concentration of Cd^2+^ was 1 × 10^−4 ^M. The concentrations of Pd^2+^, Fe^3+^, and Cu^2+^ were 4 × 10^−6 ^M. The concentration of Hg^2+^ was 6 × 10^−7 ^M.

To study the selectivity of the system, we investigated the influences of common biological metallic ions on the fluorescence intensity of NAC-capped CdTe QDs. As shown in Figure 3, most common metallic ions, including Li^+^, Na^+^, K^+^, Ca^2+^, Mg^2+^, Zn^2+^, Cd^2+^, Pd^2+^, Fe^3+^, Pb^2+^, and Cu^2+^, exhibit no significant effect on the fluorescence intensity of QDs even at relatively high concentrations, while Hg^2+^ exhibits strong quenching ability to the intensity even at low concentration. In particular, with the addition of Hg^2+^ ions, there appears a new emission peak at the NIR region 700–900 nm, and no other metallic ions produce the similar phenomena. Different from other traditional fluorescent probes which only rely on the fluorescence change of the QDs, the newly appearing PL peak ensures excellent selectivity in detection of Hg^2+^ ions.

**Figure 4:**
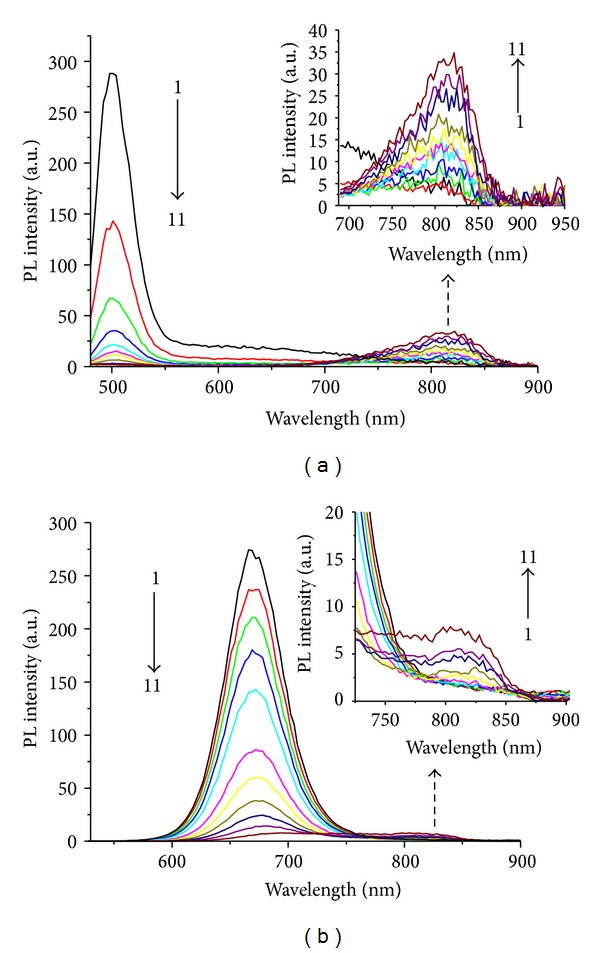
Effect of Hg(II) ions on the PL spectra of CdTe QDs ((a) *λ*_em_ = 502 nm, (b) *λ*_em_ = 670 nm) with the concentration of Hg^2+^ at 0.0, 0.4, 1.2, 2, 3, 7, 11, 15, 19, 27, and 35 × 10^−7^ M (from 1 to 11). The inset depicts the emergence of an NIR emission band on the addition of Hg(II) ions.

Furthermore, a series of different-sized QDs were chosen to study the impact of QDs diameter upon the position of the new peak. As shown in Figure 4, the newly appeared peaks appear at the relatively fixed position regardless of the difference of QDs diameters, showing that the wavelength of the new peak is irrelevant to the size of QDs. However, the smaller-sized QDs can be more easily quenched by Hg(II) ions, and the intensity of newly appeared peak is stronger, which is beneficial to the excellent sensitivity of the system. It is known that the fluorescence intensity of smaller-sized QDs can be easily influenced by the environment and other interfering materials, greatly limiting the selectivity of the system. Therefore, to ensure both good sensitivity and selectivity, this paper chooses NAC-capped CdTe QDs with their emission peak at 565 nm.

**Figure 5:**
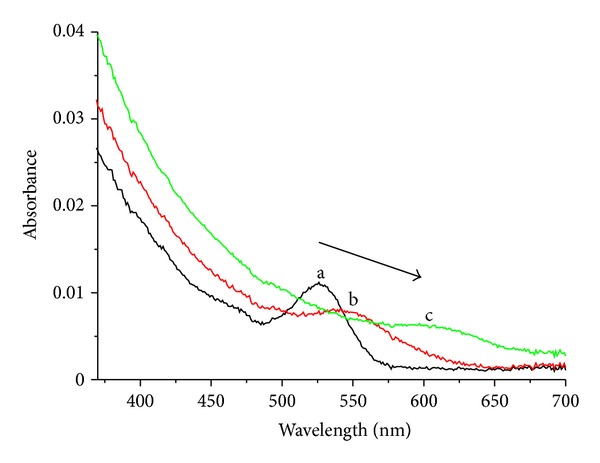
UV-visible absorption spectra of 30 *μ*M NAC-capped CdTe QDs (2.93 nm) in the presence of various concentrations of Hg(II) ions: (a) 0.0, (b) 1.0, and (c) 2.0 *μ*M.

To explain the difference in linearity of the system in low and high Hg(II) concentrations, we proposed two interaction modes between NAC-capped CdTe QDs and Hg(II) with the increase of Hg(II) ions. When Hg(II) ions concentration is relatively low (<430 nM), the Hg(II) ions interact with the carboxylate moiety of ligand (NAC) on the surface of CdTe QDs by electrostatic forces. When the concentration further increases (>430 nM), Hg(II) ions would further interact with CdTe QDs, displacing the Cd in the CdTe to form alloyed Cd_*x*_Hg_1−*x*_Te NCs [24]. These surface changes of NAC-capped CdTe QDs, on one hand, increase surface defects of QDs and render luminescence quenching of QDs; on the other hand, by facilitating nonradiative e^−^/h^+^ annihilation acting as electron-hole recombination centres and decreasing radiative e^−^/h^+^ annihilation (luminescence) [25], these alloys Cd_*x*_Hg_1−*x*_Te NCs lead to superquenching of the original QDs. Meanwhile, the formation of Cd_*x*_Hg_1−*x*_Te NCs increases the original diameter of NAC-capped CdTe QDs and narrows the band gaps of these NCs [26], producing an obvious bathochromic shift of the PL spectrum at 700–900 nm. Similar phenomenon was also reported in the paper of Liang et al. in which the formation of ultrasmall particle Ag_2_Se on the surface of CdSe QDs was proved after the addition of Ag, during their study of functionalized CdS QDs as selective Ag probe [11].  Figure 5 displays the absorption spectra of NAC-capped CdTe QDs with the addition of Hg(II) ions, in which the absorption peak red-shifts with the increase in Hg(II) concentration, inferring the formation of larger Cd_*x*_Hg_1−*x*_Te NCs.

**Figure 6:**
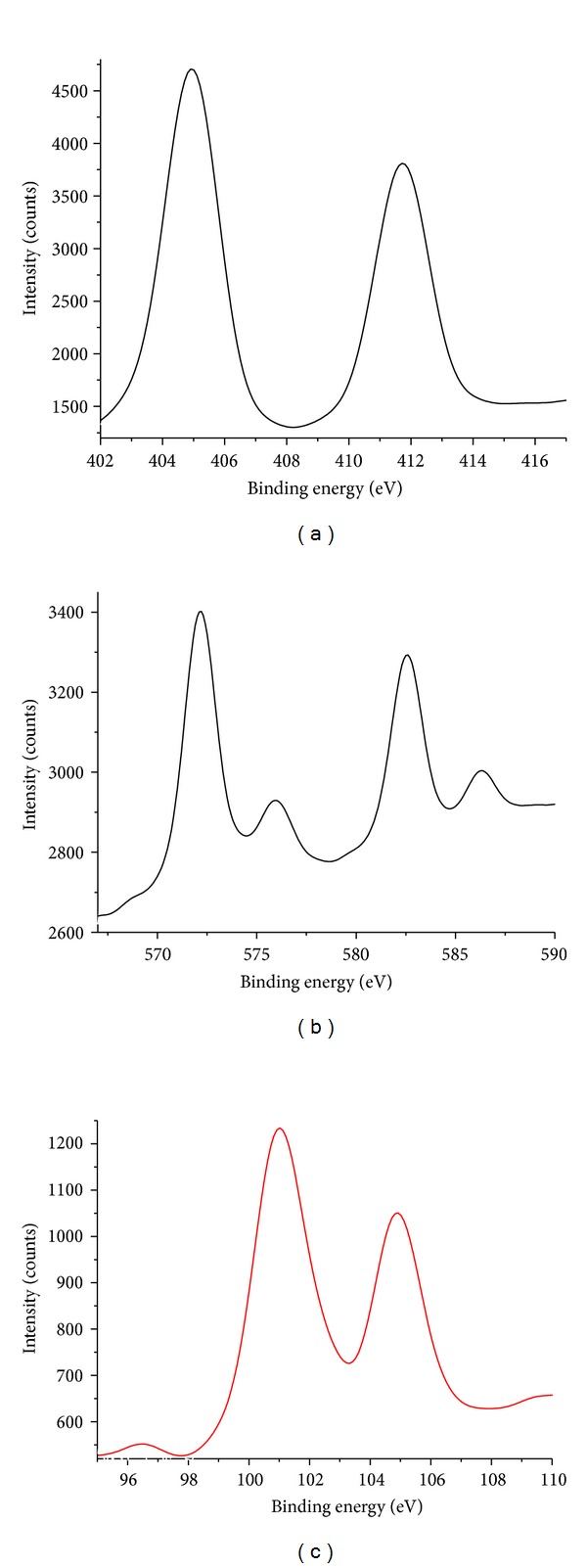
XPS of NAC-capped Cd_*x*_Hg_1−*x*_Te NCs: (a) Cd (3d), (b) Te (3d), and (c) Hg (4f).

To further confirm our proposition, XPS was used to analyze the surface components of QDs after the addition of Hg(II) ions. XPS is known as a surface analytical tool that is sensitive to the atomic composition of the outermost 10 nm of a sample surface [27]. A larger amount of Hg(II) ions was added into QDs solution, and the resultant product was precipitated by cold 2-propanol. The product was washed and dried overnight for XPS measurement. If Hg(II) ions combine only with the ligands on the surface of NAC-capped QDs, they will be removed by the addition of 2-propanol, while if Hg(II) ions bind directly onto the surface of CdTe QDs and form alloyed Cd_*x*_Hg_1−*x*_Te NCs, they will be precipitated out. The appearances of characteristic Cd (3d_5/2_) peak at 404.8 eV and Te (3d_5/2_) peak at 572.2 eV are depicted in Figure 6. The typical binding energies for the Hg (4f) peaks at 101.0 and 105.0 eV confirm the presence of Hg on the Cd_*x*_Hg_1−*x*_Te NCs (Figure 6(c)). 

## 4. Conclusion

The selective detection of Hg(II) ions through the interaction of Hg(II) ions and NAC-capped CdTe QDs has been realized in our system. The fluorescence intensity of CdTe QDs can be remarkably quenched by the addition of Hg^2+^ ions, and there appears a new peak at about 820 nm. The linear response range and the limit of detection are 20–430 and 8.0 nM Hg(II), respectively. The influence of most physiologically important metallic cations upon the system, including Li(I), Na(I), K(I), Ca(II), Mg(II), Zn(II), Cd(II), Pd(II), Fe(III), Pb(II), and Cu(II), was tested to prove the selectivity of the system. We further proposed that the possible mechanism for the new peak is that Hg(II) ions displace the Cd in the CdTe and form alloys Cd_*x*_Hg_1−*x*_Te NCs, which has been proven by ultraviolet absorption spectrum and X-ray photoelectron spectrum.
